# Focus on the Gut–Kidney Axis in Health and Disease

**DOI:** 10.3389/fmed.2020.620102

**Published:** 2021-01-21

**Authors:** Elisavet Stavropoulou, Konstantia Kantartzi, Christina Tsigalou, Theoharis Konstantinidis, Gioulia Romanidou, Chrysa Voidarou, Eugenia Bezirtzoglou

**Affiliations:** ^1^Centre Hospitalier Universitaire Vaudois (CHUV), Lausanne, Switzerland; ^2^Department of Infectious Diseases, Central Institute, Valais Hospital, Sion, Switzerland; ^3^Department of Medicine, Democritus University of Thrace, Alexandroupolis, Greece; ^4^Hospital “Sismanoglio”, Komotini, Greece; ^5^Department of Public Health P.U., Arta, Greece

**Keywords:** gut, kidney, gut–kidney axis, microbiome, health, disease

## Abstract

The recent new developments in technology with culture-independent techniques including genome sequencing methodologies shed light on the identification of microbiota bacterial species and their role in health and disease. Microbiome is actually reported as an important predictive tool for evaluating characteristic shifts in case of disease. Our present review states the development of different renal diseases and pathologies linked to the intestinal dysbiosis, which impacts on host homeostasis. The gastrointestinal–kidney dialogue provides intriguing features in the pathogenesis of several renal diseases. Without any doubt, investigation of this interconnection consists one of the most cutting-edge areas of research with potential implications on our health.

## Introduction

Newborns' intestinal colonization established either by vaginal delivery or by cesarean section has been extensively studied (1, 2). Multiple factors such as immune system, diet, environment, and genetic endowment are involved in the development of the newborn's intestinal microbiota. Moreover, other endogenous and exogenous factors such as hospital personnel, infections, stress, vaccination, personal habits, hormonal status, and age (3–5) are entailed in the processes of the microbiome establishment. To these issues, new technological breakthroughs such as next-generation sequencing of the 16S ribosomal RNA gene (rRNA) and metagenomics whole-genome shotgun sequencing advanced and enlightened our knowledge on the intestinal microbiome and the barrier effect repulsing pathogenic bacteria from gut colonization. The 16S rRNA technologies provided taxonomic resolution of bacterial communities at species and strains level (6). Gut bacterial communities participate dynamically in the metabolism and immune system education and erect potent positive or negative interconnections between bacteria and other systems. These interconnections are bidirectional and reported collectively as the gut–kidney axis. The fecal microbiota composition and functionality seem to play a crucial role in the homeostasis but also in the development of kidney diseases. Although there is a knowledge gap on the different interplay components between gut microbiome and organs that needs to be completely clarified, hopefully technological advancements can permit us to better appreciate this multifaceted issue (6, 7). Our present review promotes recent knowledge that certainly will stimulate more study and research on the involved complicate pathways of the gut–kidney axis.

### Summarizing the Gut Microbiome

As known, there is a plethora of studies on the human gut microbiome as it is one of the richest in population microbiomes of human body (8–11). Without any doubt, the gut keeps a key role in nutrients absorption and substances synthesis such as vitamins, amino acids, and enzymes, as well as production of short-chain fatty acids (SCFAs) (12). Acetate, butyrate and propionate are SCFAs coming from bacterial carbohydrate fermentation, and they represent energy sources in the colon (13). Specifically, they enhance epithelial integrity by providing energy to epithelial cells, they participate in the immunomodulation processes, and finally they act as a shield against pathogenic bacteria (13). Two main signaling mechanisms are taken through SCFAs (14): the activation of G-protein–coupled receptors (GPCRs) and the inhibition of histone deacetylases (HDACs) (14). GPCRs are receptors of SCFAs and involved in the processes of metabolism, inflammation, and disease generally. Yet, the free fatty acid receptor-2 and−3 (FFAR2 and FFAR3) where SCFAs are activated are located in multiple human sites including gut and participate in the regulation of metabolism (15). Yet, SCFAs affect the physiology of the gut epithelial cells by inhibition of HDACs leading to chromatin remodeling and shifts in transcription processes (16). HDACs showed antiproliferative and anti-inflammatory outcomes *in vitro*– and *in vivo*–developed models of inflammation (16).

As defined recently (17), the intestinal microbiome is composed by microorganisms, bacteria, viruses, protozoa, and fungi, as well as of their genetic equipment. It is believed that the human gut microbiome contains more than 3.3 million prokaryotic genes (18).

To this point, the Human Microbiome Project in the United States [US National Institutes of Health (NIH), http://commonfund.nih.gov/hmp/] (19) and the metaHIT Consortium (20) in Europe have been able to characterize the composition of bacterial communities at the various human ecosystems. In addition, the MetaHIT project classified bacterial populations in three different enterotypes (21). Initially, those projects were focused on the gut intestinal microbiotas and other open human sites due to the facility in collecting samples. Enterotype 1 features by high *Bacteroides* levels and enterotype 3 by high *Ruminococcus* levels. Enterotype 2 showed important *Prevotella* population and few *Bacteroides*. Yet, these enterotypes are not influenced by age, ethnicity, gender, and body weight (22). Diet seems to be a crucial factor to the gut microbiome identity. Western diet plenty of proteins and fats showed domination of *Bacteroides*, whereas diet rich in carbohydrates and fibers of *Prevotella* species (22).

In short, the gut microbiota contains four bacterial groups called *phylum*, which are *Firmicutes, Bacteriodetes, Actinobacteria*, and *Proteobacteria* (23).

Notably, the evolution of the gut microbiome and its shifting were extensively studied from newborn to the adult age (1–3, 8–10). Multiple factors influence the magnitude of the bacterial mosaic, which is in perfect balance in healthy people in a state called *symbiosis* (4, 5). Stress, feeding, environment, hormones, and genetic predisposition are stated as important determinants (4, 5, 11). However, when disruption of this balance occurs, dysbiosis is installed, which is shown to be closely associated with disease (23–25).

### The Urinary Microbiome in Health

Although our subject is not based on the role of the urinary microbiome, we included this part of knowledge as wiping back to front the urinary tract is in close continuity with the kidney.

The study and the importance of the urinary tract microbiome aroused interest during recent years (26, 27). In contrast to the old belief that the urinary tract is sterile (28) recent studies demonstrated that the urinary tract possesses a unique microbiota (28) with a dynamic role in the maintaining of urinary health due to its metabolic capacity.

The urinary system is constituted of the kidneys, ureters, the urinary bladder, and the urethra. Ascendant microbial populations following lower urinary tract infections as bladder cystitis may cause kidney infections.

The kidneys are the seat of the urinary system primary functions as they are the site of blood filtering, electrolyte balance, and maintaining water. Kidney is considered sterile in both men and women. Bacteria found at sites distal to the kidney, bladder, and urethra seem to influence the urologic health in multiple ways. Besides, the gut microbiome carries complex and dynamic microbiota influencing human physiology, nutrition, and immunity. When disruption of this microbiota occurs, microbial communities are imbalanced, and there is a rupture of the interconnection between these microbial communities and human cells, resulting in health disorders.

It is of note that the urinary tract was not entered initially in the global Human Microbiome study as it was considered immoral and unethical to proceed to bladder biopsies or suprapubic aspirates from healthy persons in order to characterize (26, 28) the microbiota composition. *Lactobacillus* and *Streptococcus* were the genera most frequently reported in the urinary microbiota (26).

*Klebsiella, Rhodanobacter, Saccharofermentans, Jonquetella, Alloscardovia, Burkholderia*, and *Veillonella* were also isolated from the urinary microbiota (26). It is of note that *Parvimonas, Jonquetella, Saccharofermentans*, and *Proteiniphilum* genera were found in individuals older than 70 years (29).

Surprisingly, some bacteria genera were detected only by cultures (*Trueperella*), whereas other genera only *via* sequencing methods (*Atopobium*) (30). Methodologies limitations should explain these differences as several bacterial strains are not growing in expanded quantitative urine culture (27), and also the 16S rRNA sequencing is not distinguishing between living and dead bacteria (31). Nevertheless, scientists underpin the importance of 16S rRNA sequencing as it combines presence of living and dead bacteria as an imprint of the microbes that once prevailed in the microbiota (19). Furthermore, collection of middle stream urine, which is considered overall sterile, using the 16S rRNA sequencing, showed (32) predominance of *Lactobacillus* in women and *Corynebacterium* in men. Certainly, the above results are at least expected, due first to the shortness and proximity of the female urethra to the vagina where important populations of *Lactobacillus* inhabit and second to the anatomic structure of the male genital system, which is housed by *Corynebacterium* coming probably from the skin microflora (33).

### The Gut–Kidney Axis

As discussed previously, when microbial communities are imbalanced, the disruption of the normal gut microbiota may lead to intestinal dysbiosis due to the breaching of the intestinal barrier. Moreover, it is reported that passage of viable bacteria may occur from the gut to other extraintestinal sites including the kidney. This bacterial translocation may be associated with bacterial dysbiosis, bacterial overgrowth, and low host immune defense (34–36).

The gut microbiota produces many uremic solutes and toxins, such as indoxyl sulfate, *p*-cresyl sulfate (PCS), and trimethylamine (TMA) N-oxide during chronic kidney disease (CKD). Yet, increasing urea concentration leads reciprocal to the intestinal microbiota alteration (37). Uremic toxins may cause renal anemia, pruritus, fatigue, mineral bone disorder, neurological damage, and cardiovascular impairment in CKD patients (37).

**Figure 1:**
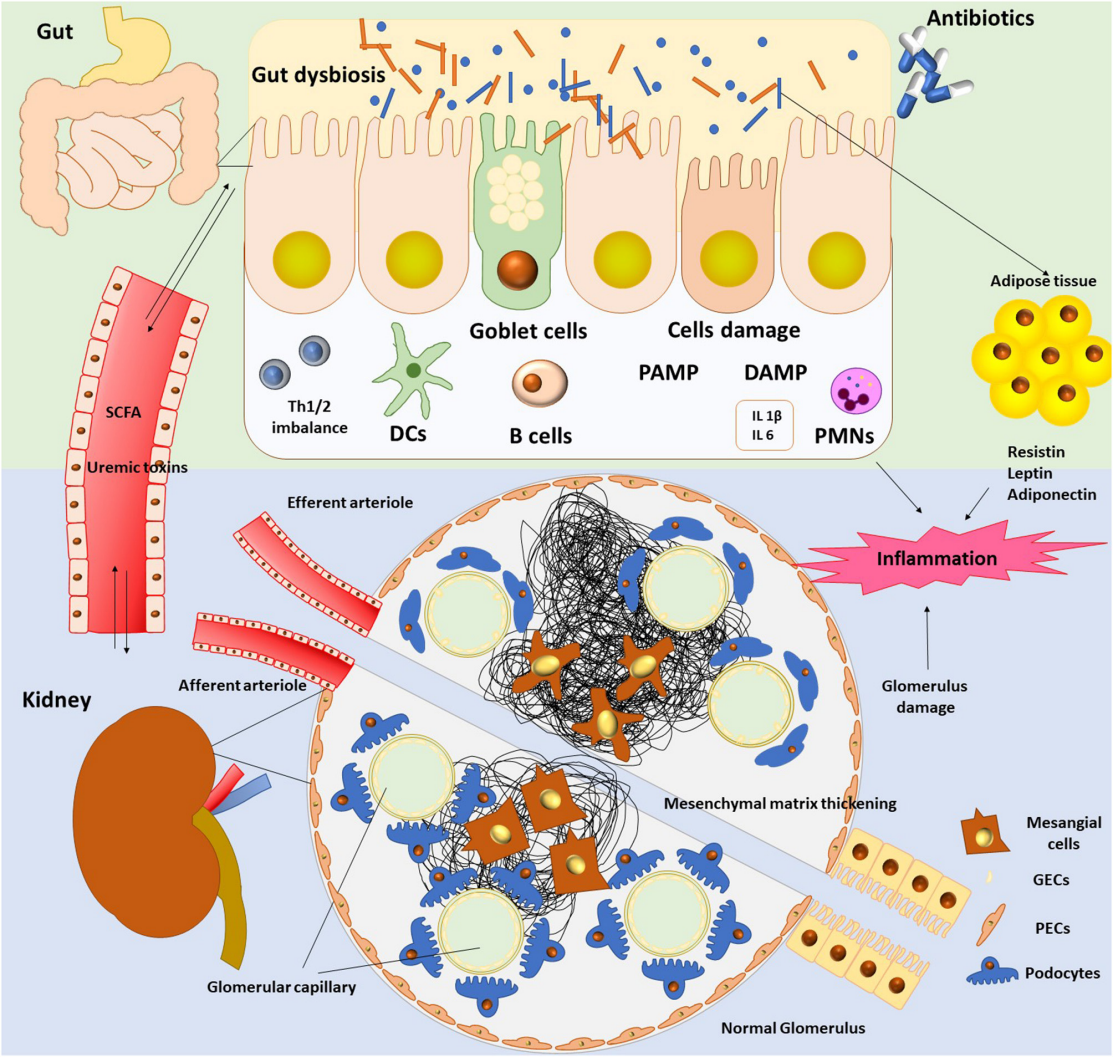
The gut–kidney axis.

The pathogenic interconnection between gut microbiota and kidney diseases is called the gut–kidney axis (Figure 1) and seems to be implicated in a wide range of clinical manifestations such as CKD, acute kidney injury (AKI), hypertension, nephrolithiasis, immunoglobulin A (IgA) nephropathy, hemodialysis, and peritoneal dialysis (7, 37).

Technological advances in metagenomics–metabolomics reveal the contribution of the gut- kidney axis on different kidney pathologies as they help us to get a more comprehensive knowledge of the microbiome. However, the underlying mechanisms between gut microbiome and host in health and disease remain obscure. Therefore, the know-how of this interconnection may clarify disease etiologies and pathogenesis.

### Metabolic and Immune Pathways Involved in Kidney Diseases

Gut microbiota has deployed a holistic shield system, which assignment is either to identify and attack the aggressors or to develop mechanisms allowing settlement of damaging (3). In this vein, the host immune system plays a crucial role to preserve the microbial intestinal balance *via* the barrier effect (3). Bacteria of our microbiome are tightly attached to the gut mucosa and inhibit colonization by pathogenic bacteria; this is the “colonization resistance.” This is a biological barrier, and bacteria support themselves by producing antimicrobial proteins (AMPs) ***via*** Paneth cells, either by yielding SCFAs (12, 16). Notably, the gut microbiome through its intestinal barrier regulates homeostasis and function of both innate and adaptive immune systems locally and systemically (38). Moreover, a physical barrier seems to be evident in the barrier concept, as gut epithelial cells through apical tight junctions (TJs) transmembrane proteins form a shield preventing free diffusion from lumen to **lamina propria** (3, 14). The gut microbiota is reported to be in strait relation with the mucosal immune system (14) as gut bacteria and their toxins can cross this mucosa and spread in the bloodstream, tissues, and organs when this barrier is breached (3).

Last but not least, the activation of an immune barrier is taking place in order to preserve homeostasis (3, 14).

When barrier is ruptured “leaky gut,” immune cells are activated, infiltrate the kidney, and induce proinflammatory and anti-inflammatory functions, as well as regulatory signals to modulate the neutrophil response (39). Neutrophils and macrophages are implicated in the innate immunity as first-line response of non-specific defense against pathogens (40). Decrease of the phagocytic capacity of macrophages impacts negatively the kidney function and produces a chronic inflammation status (40). Innate immunity keeps a crucial potential in many renal diseases (41).

The key role of the pattern recognition receptors (PRRs) in the innate immune response and their expression during inflammation was revealed (42). Notably, monocytes, neutrophils, and macrophages amplify PAMPs (pathogen-associated molecular patterns) and DAMPs (danger- associated molecular patterns) (43) triggering immune response. Scientists have studied PRRs with a special focus on TLRs (Toll-like receptors), which are membrane glycoproteins (42). TLRs are expressed in renal cells inducing activation of mitogen-activated protein kinases, nuclear factor-κB (NF-κB), and activator protein-1 (44, 45).

It is of note that PRRs can be involved in the exacerbation of infection-mediated renal disease, but equally to lupus nephritis by recognition of nucleosomal autoantigens (46).

Renal tubular epithelial cells are also participants in immunity by production of chemokines, cytokines, and antimicrobial compounds (47). These cells keep an important role in inflammation processes as they could regulate positively or negatively T-cell responses as they express costimulators of T cells (ICOS-L) and B7-H1 molecules (48).

Likewise, dendritic cells (DCs), macrophages, and T regulatory cells (Tregs) contribute to evoke an adaptive immune response (39). Activation of DCs results in production of proinflammatory cytokines such as interleukin 12 (IL-12), IL-6 (39). Specifically, DCs induce the differentiation of naive CD4^+^ T cells into regulatory T (Treg) cells and the maturation of B cells into IgA-secreting cells (39, 49).

Yet, there is a DC-mediated recruitment of Tregs, depending on the activation of alternate autophagy pathways (50). Treg recruitment seems to be a strategic point to protect from inflammation and amplify homeostasis by boosting microbiome (51).

In assistance of the above, the activity of T helper 17 (Th17) cells, a subset CD4 T helper (Th) cells, is designated by secretion of the proinflammatory IL-17 (49, 52).

Likewise, renal tubular epithelial cells release NF-κB, which controls proinflammatory response (53).

To this end, the innate lymphoid cells (ILCs) act by tampering macrophage production of the proinflammatory cytokines IL-1β, IL-12, IL-23, IL-22, and interferon γ (49, 54). In this light, it is shown that the aryl hydrocarbon receptor of IL-22 in ILC response (ILC3) contributes to the extinction of inflammatory Th17 cell responses that maintains Treg-mediated gut homeostasis (55). Moreover, the suppression of Th17 cells in the gut promotes their translocation and activation in kidney (49). Intestinal Th cells can be activated in the kidney via a CCL20/CCR6 axis (56).

The gut microbiota interacts by means of microbial-associated molecular patterns (MAMPs) or SCFAs, as well to reduce inflammation in kidney (57). Expression of 4 receptors (GPR41, GPR43, Olfr78, and GPR109a) is found in kidney by reverse transcriptase–polymerase chain reaction (PCR) (58) associated with distinct pathological states (59, 60). SCFAs affect upon kidney injury by regulating inflammation (57). Administration of SCFAs in animals seems to decrease levels of reactive oxygen species, as well as cytokines production (61). Additionally, activation of NF-κB was suspended in renal epithelial cell and low amounts of mRNA of the TLR4 were found in animals undertaken SCFAs (49, 61). Finally, maturation of DCs was also suspended, and differentiation of naive CD4+ T cells into Treg cells, as well as the maturation of B cells into IgA-secreting cells, was inhibited (49, 61). To this end, SCFAs modulated the hypoxia effects produced in renal epithelial cells by boosting biogenesis of mitochondria, and thus, their possible role as a new therapeutic agent should be challenging (49, 61).

Moreover, as stated, production of AMPs and IgA by Paneth cells in the gut contributes to the host microbiota balance (62, 63).

Lactobacillales in the gut can boost Treg cells and damper disease-causing Th17 cells in kidney of lupus mice models in order to impair inflammation (64). Owing to the above, strategies focusing on the microbiota modulation could be challenging.

### Microbiota and Disease States

#### Chronic Kidney Disease

CKD is the loss of kidney function, and it is a severe health issue. Progressive loss or failure of kidney function has an effect on the blood concentration of different noxious substances (65), which are usually metabolized and excreted by the kidney. These substances, as well as high urea concentrations, accumulate in blood and causes uremia (65). Several gut bacteria possessing ureases are able to convert urea to ammonia.

This conversion leads to shifts of the luminal pH, resulting in uremic enterocolitis. However, there is a reciprocal process as high urea concentration enhances urease-producing bacterial growth. Heavy urea concentrations modify the biochemical environment of the intestine.

Researchers evaluated the gut microbiome in relation to the kidney disease and the development of kidney stones (66) (Table 1). Adults with kidney disease housed a microbiota characterized by an enhanced number of *Enterobacteriaceae* and *Streptococcaceae*, in contrast to the dropping populations of *Prevotellaceae* and *Roseburia* (23). Concerning adults developing kidney stones, a varied microbial community of *Bacteroides, Enterobacter, Lachnospiraceae NK4A136 group, Christensenellaceae, Ruminiclostridium 5 group, Dorea*, and its genus *Christensenellaceae R7 group* is present (66).

**Table 1:** Shifts of bacterial taxa in certain kidney or kidney-associated diseases.

**Health disorder**	**Population increase**	**Population decrease**
Chronic kidney disease (CKD)	*Bacteroidetes, Proteobacteria, Actinobacteria*	*Firmicutes*
Kidney stones presence	*Bacteroidetes, Firmicutes*	
End-stage renal disease (ESRD) hemodialysis	*Proteobacteria, Firmicutes, Actinobacteria*	
ESRD peritoneal dialysis	*Firmicutes, Actinobacteria*	
Obesity	*Bacteroidetes, Proteobacteria*,	*Firmicutes*
Immunoglobulin A nephropathy	*Actinobacteria, Proteobacteria*,	
Diabetes	*Bacteroidetes, Proteobacteria*	*Firmicutes, Actinobacteria*
Transplanted patients	*Proteobacteria, Bacteroidetes (acute rejection)*	*Firmicutes, Actinobacteria*
Autoimmune disorders	*Bacteroidetes, Firmicutes*	*Actinobacteria*

As stated in patients with CKD, dysbiosis leads to the release of uremic toxins (67). Recent research associated gut-derived uremic toxins with the progression of CKD, cardiovascular disease, and mortality (68). In fact, higher numbers of uricase and urease-producing bacteria as well as indole- and *p*-cresyl-forming enzymes bacteria are found in subjects with end-stage renal disease (ESRD) (37). *Proteobacteria, Firmicutes*, and *Actinobacteria* taxa are observed in high numbers at the ESRD (69).

CKD breaches the balance between normal microbiota and pathogens and permits pathogens' overgrowth. As a result, breakdown of the intestinal barrier occurs because of the rupture of the epithelial tight junctions (ETJs). ETJs are protein complexes that prevent leakage of solutes and water by forming selective channels (70). Thus, there is loss of the intestinal permeability permitting bacteria to translocate to other organs (34). Evidence in uremic rats showed bacterial translocation across the intestinal wall in the mesenteric lymph nodes (71).

Moreover, lipopolysaccharides (LPSs) originating from the cell wall of Gram-negative bacteria increase the gut TJ permeability by enhancing the enterocyte membrane TLR4 and CD14 expression (72). Additionally, LPS could mobilize the innate immune cells through TLR4 and NF-κB pathways (72). As inflammation, oxidative stress and impairment of the immune response occur; there is release of inflammatory cytokines due to the stimulation of DCs by pathogens and activation of the Th17/Th1 T-cell response (49, 53, 72).

In the intestine, there is prevalence of anaerobic bacteria that are metabolizing in absence of O_2_, a process called fermentation, the different substances imported with food. In fact, there is fermentation of the amino acids' tyrosine to *p*-cresol and tryptophan to indole (73).

Those compounds should be further metabolized in the liver to PCS and *p*-indoxyl sulfate (IS) and TMA n-oxide (TMAO) before circulating free or serum proteins—binded (73). The above noxious uremic toxins are eliminated by tubular secretion in the kidneys; their increased levels are indicative of renal failure and progression of CKD (73). These toxins have deleterious effects on different body tissues as renal tubular cell damage, coagulation disturbances, leukocyte activation, endothelial dysfunction, cardiac fibrosis, cardiac hypertrophy, cardiovascular disease, cardiovascular mortality, atherosclerosis, insulin resistance, and reduction of fat mass (73). It is of note also that due to the prevailing oxidative stress in osteoblasts, IS impedes bone formation, which contributes to metabolic bone disease (74).

Therefore, because of the dysbiosis (37), the beneficial microbiota dominated by *Bifidobacterium* and *Lactobacillus* disappear gradually, and a dropping in SCFAs and bile acid levels is observed due to the microbiota shifting and pathogen dominance (75). SCFAs and specifically butyrate are important energy sources for colonocytes (76) and also play an important role in the epithelial integrity. Moreover, activation of the SCFA receptor GPR109A is linked to the suppression of several proinflammatory mediators (76).

Authors reported (77) decrease in the anaerobic microflora in patients with CKD, while an increase of the aerobic microflora background (78) is observed with a predominance of *Enterobacteriaceae* (79). As previously discussed gut microbiota in healthy recipients possess three main enterotypes (80): *Bacteroides, Prevotella*, or *Ruminococcus*. As previously stated, the gut microbiota in patients with CKD is shifted, characterized by low numbers of *Lactobacillaceae* and *Prevotellaceae* families and higher Enterobacteria and Enterococci (79).

CKD and ESRD influence considerably the composition of the intestinal flora and by extension its functions due to the multifaced occurring processes: impact of uremia, inflammation, and diet (79). However, it is noteworthy that dysbiosis in CKD patients may enhance uremic toxin concentrations that in their turn are involved in the progression of CKD (79). In developing countries, CKD is related to hypertension, diabetes, obesity, cardiovascular disease, glomerular and tubule interstitial, and aging (68). Moreover, exposure to factors such as toxins, antibiotics, drugs, iron intake, reduced dietary fiber intake, increased protein absorption, and finally slow intestinal transit could induce CKD and aggravate complications (68).

#### Dialysis Patients

When irreversible renal failure occurs, ESRD could be treated by hemodialysis or peritoneal dialysis in order to eliminate the toxic solutes or finally renal transplantation following availability of compatible donors. Dialysis permits elimination of a large number of toxins and waste solutes involved in the uremic syndrome. Of note is that hemodialysis or peritoneal dialysis may augment the permeability of the intestinal barrier in CKD patients and thus contributing to the translocation of endotoxins (79).

The gut microbiome of ESRD adult patients (Table 1) who underwent hemodialysis showed rise in *Proteobacteria, Actinobacteria*, and *Firmicutes* with preponderance of the subphylum *Clostridia* (79). Differences were observed in pediatric patients who underwent hemodialysis, as an increase in *Bacteroidetes* was found and reduction in *Proteobacteria* taxa (80). Besides, dialysis patients have low levels of SCFAs and butyrate due to the modification of the intestinal milieu and dysbiosis (81). Peritoneal dialysis patients' house differences in their gut microbiota, as a decrease in the taxa *Firmicutes* and *Actinobacteria* is observed.

A global study including more than half a million patients during 7 years in the United States states that the peritoneal dialysis was more frequent in rural areas (82). Surprisingly, the higher mortality rates were registered among Hispanic white patients living in remote rural places (82). However, people living in distant communities showed higher prevalence of ESRD to support a dialysis unit (82) and were supposed often to have a transplant (82). Likewise, when comparing dialysis patients in United Kingdom living in urban and rural areas, mortality rates were higher for people living in industrial areas (83). Yet, transplant rates were lower in Native Americans living in rural areas (82).

Overall, transplantation could impact on changes on the urinary and gut microbiota (84). Moreover, genetics, epigenetics, pharmacogenetics, hormonal status, and environmental factors (85) seem to impact and gut microbiota to be associated to worsen the situation in kidney allograft receivers (84). As stated, there is a reciprocal dialogue between kidney and gut, which is supposed to have a dynamic stress impacting on the microbiota. In this light, host immune responses initiate and could lead to infection and allograft rejection (84).

The importance of the microbiome is previously stated. Gut microbiota is able to trigger antigen-presenting cells (APCs) to initiate immune responses and alloimmune reactivity as observed in allogeneic bone marrow transplantation (HSCT) (86). When these allograft recipients were supposed to have gut decontamination, acute graft-vs.-host disease declined (87). Research showed that posttransplant rejection is closely linked to the immunosuppression-driven dysbiosis (87). In this vein, overgrowth of opportunistic microorganisms and *Escherichia coli* is observed (88), as well as decreased diversity (89). Dysbiosis and bacterial diversity seem to be more pronounced when postoperative complications happen (89, 90), as dropping amounts of *Firmicutes* phylum and *Lactobacillales* order confluence together with enhancement of the *Proteobacteria* phylum population (91). Important changes in microbiota composition can be observed 1 month after transplantation linked basically to infectious events (87). Yet, enterococcal infections and diarrhea are associated with posttransplantation gut microbiota shifts (87).

*Bacteroides, Streptococcaceae, Enterobacteriaceae*, and *Bifidobacteriaceae* were found increased in cases of acute cell rejection, whereas *Lactobacillaceae, Ruminococcaceae, Clostridiaceae*, and *Peptostreptococcaceae* showed increased amounts in successful cases of transplantation (92). Interestingly, patients hosting *Faecalibacterium prausnitzii* in their microbiota seems to be in need of higher tacrolimus therapeutic doses (93), highlighting also the important role of the microbiota on drug metabolism as bacteria possess CYP P450 enzymes involved in drug metabolism (94).

#### Autoimmune Disorders

Gut dysbiosis may impact and promote autoimmune disorders such as inflammatory bowel disease (IBD), rheumatoid arthritis, type 1 diabetes, and multiple sclerosis. However, little information is obtained concerning the systematic lupus erythematosus (SLE), lupus nephritis (46, 64, 95), and intestinal microbiota (49).

The increased numbers of *Helicobacter pylori* antigen found in renal biopsies argue that bacteria may keep an impact in membranous nephropathy and lupus nephritis (96).

Therefore, the gut microbiota amending polarization of the T-cell subsets and natural killer cells may have important immunomodulatory outcomes upon the autoimmune kidney disease (96). Immune system's impairment may also induce profound kidney injury (97).

Researchers stated that individuals with Crohn disease have a decrease in the abundance of *Firmicutes* and an increase in *Bacteroidetes* (98) (Table 1). Yet, they found that the enzyme urease shifts the microbiota in IBD rat models. Several intestinal bacteria possessing ureases convert urea to ammonia (99). Ammonia, a source of nitrogen, is utilized for protein synthesis in the host by hepatic metabolism. However, in individuals with liver damage, this conversion may be noxious, as high circulating ammonia levels result in hepatic encephalopathy (100).

The importance of the gut microbiota in the pathogenesis of renal impairment in lupus is stated (64). Scientists developing a lupus nephritis model in MRL/lpr mice observed considerable decrease of *Lactobacillales* in the gut microbiota and a leaky gut (101).

Engineering the gut microflora with bacteria having low urease activity in mice, it was demonstrated that ammonia levels were dropping, as well as neurobehavioral effects and mortality (102).

In SLE patients, a decrease in the ratio *Firmicutes/Bacteroidetes* registered was studied by two different methodologies: 16S rRNA gene-based analysis and quantitative PCR, whereas *Lachnospiraceae* and *Ruminococcaceae* were associated with healthy individuals (103). Higher amounts of *Bacteroidetes* were found in SLE patients (103). Therefore, glycan degradation is overexpressed in the microbiota of SLE patients, presumably due to the higher population of *Bacteroidetes* (104). It is noteworthy that oxidative phosphorylation processes seem to be associated with SLE patients (105). T cells from patients with active lupus enhance mitochondrial oxidative phosphorylation, effecting in O_2_ generation that amends proteins (105).

The Goodpasture syndrome or anti–glomerular basement membrane disease (106) is a rare autoimmune disease that affects both the kidneys and the lungs. In kidney, the disease is due to circulating autoantibodies against the domain of the α3 chain of type IV collagen of glomerular and alveolar basement membranes. As a result, the patients develop rapidly glomerulonephritis with alveolar hemorrhage (106).

According to the hygiene hypothesis, early exposure to bacteria amends kidney damage and inflammation (107). In fact, germ-free mice models with ischemia-induced.

AKI showed extended renal damage compared to the control animals due to the Th1-type response as in the autoimmune disorders (107).

#### Immunoglobulin a Nephropathy

Immunoglobulin A nephropathy (IgAN) is a type of primary glomerular disease in adults (108). Prior studies have demonstrated an association between IgAN and dysregulation of the gut-associated lymphoid tissue (109). Gut microbiota shifting (Table 1) and dysbiosis may be the angular stone of the IgAN (60) as supported on a cross-sectional study in Chinese patients.

IgAN patients showed an increased abundance of *Fusobacteria*, whereas a decreased abundance of *Synergistetes* was stated (110). *Hungatella, Escherichia-Shigella*, and *Eggerthella*, genera having a pathogenic potential, were found in IgAN individuals, whereas the genus *Escherichia-Shigella* was linked pragmatically to the urinary albumin-to- creatinine ratio (uACR) but in a negative manner with glomerular filtration rate. Therefore, the genus *rectale_group* was found in low numbers in the IgAN patients associated negatively with the urinary uACR (110). Moreover, it was found that levels of urinary metabolites such as free amino acids and organic volatile compounds vary significantly between the progressor and non-progressor IgAN patients (111).

Chronic bacterial colonization and chronic infections of the upper respiratory tract may be involved in the development of IgA vasculitis and IgAN (112). Additionally, recent studies reported an association individual of IgA alimentary antigens, particularly gliadin and gut- associated hyperreactivity lymphoid tissue in IgAN patients (109).

#### Diabetes

Diabetes mellitus (DM) is characterized by metabolic disorders, high blood sugar concentrations, and frequently inflammation. DM is involved in CKD and ESRD.

DM type 2 (T2DM) is a metabolic disease characterized, among others, by inflammation as an outcome of visceral obesity, but when CKD or ESRD disease on dialysis is taking place, the inflammation status is multifaceted (113).

The interplay between increased intestinal permeability, high levels of LPS, and intestinal dysbiosis is known as endotoxemia and predisposes patients to T2DM, CKD, or ESRD on dialysis (113). During this chronic inflammation status of diabetes, there is decrease in beneficial bacteria numbers producing SCFAs and increase in proteolytic bacteria leading to uremic toxicity (113, 114).

By the aid of V4 16S rRNA pyrosequencing, it was reported that *Bacteroidetes* and *Proteobacteria* were higher in diabetic patients compared to the healthy group (114) (Table 1). In fact, the ratio between *Firmicutes* and *Bacteroidetes* decreases in human type 2 diabetes compared with controls (114), and the ratio *Bacteroidetes* to *Firmicutes* correlates positively with reduced glucose tolerance (114).

Diabetes type 1 in rats was reported to be associated with higher amounts of *Bacteroides* species (115), whereas *Bacteroides*-*Prevotella* species were related to a strong decrease of metabolic endotoxemia and inflammation in type 2 diabetes vs. class *Clostridia* and *C. coccoides*–*E. rectale* group (116).

It is noteworthy that the beneficial *Bifidobacterium* was associated with improved glucose tolerance and low inflammation (117). Surprisingly, *Lactobacillus* numbers were higher in diabetic persons compared to the non-diabetic. *Lactobacillus* have immunomodulating properties that may possibly be involved in the chronic inflammation processes in diabetic patients (118).

Diabetic kidney disease is a major cause of renal injury (119), occurring in 30% of the diabetic individuals. As stated, diabetes amends significant shifts in the dysbiotic microbiota and several metabolites (114). It was demonstrated that inhibition of the metabolite phenyl sulfate (PS) limits albuminuria in diabetic mice, whereas PS production is positively correlated with the progression of albuminuria (119).

In conclusion, there is evidence that the regulatory effect of the renal function is linked to specific bacteria of the microbiota that could modulate the renal function in diabetic nephropathy (120).

#### Obesity

Obesity is a risk factor for kidney disease as studies have shown that metabolic syndrome is also associated with the progression of kidney disease (121). Moreover, diabetes and glucose homeostasis are associated with obesity (122).

Unexpectedly, a negative correlation between ratios of *Bacteroidetes* to *Firmicutes* and body mass index (BMI) was found (114, 123) (Table 1) as it seems that obesity and diabetes are associated with distinct species of the gut microbiota. In obesity, dysbiosis is featured by an increase in the ratio *Firmicutes/Bacteroidetes* (124), and this correlates with studies reporting that weight increase is linked to a preponderance of *Firmicutes* against *Bacteroidetes* (125, 126). In line with the above, *Prevotellaceae* were also found at high levels in obese subjects, whereas *Firmicutes* were declined in patients with post–gastric bypass (127) as they were supposed to have specific diet. Moreover, dropping in *Clostridium* species, *C. coccoides* group, and increasing in *Bacteroides*-*Prevotella* in case of weight loss were observed (124). In this regard, *Firmicutes* were associated with a low-fat/high-fiber diet (128). Yet, diet including whole grain increases the ratio *Firmicutes/Bacteroidetes* (129).

Overall, a confined intervariation in the diversity of fecal microbiota was evident in diabetic patients, reflecting diet differences, habits, environmental stressors, and other factors (129, 130).

It is stated that dysbiosis may be the etiology of the childhood obesity (126) as it is believed that increased *Firmicutes*/*Bacteroidetes* ratio in obese subjects could be a consequence of chronic dysbiosis and metabolic impairment (126).

Another aspect supported by researchers argues the interconnection of metabolic diseases with the presence of Gram-negative bacteria in the gut (117, 121). As known, LPSs situated in the outer membranes of gram-negative bacteria stimulate the inflammation processes and cause endotoxemia (131). Gut microbiota in diabetic subjects is characterized by Gram-negative bacteria of the phyla *Bacteroidetes* and *Proteobacteria* (113).

Therefore, microbial balance in the human gut seems to be associated with the pathophysiology of each disorder as distinct bacterial species may be involved and determine the progression and severity of CKD disease (131, 132).

#### Hypertension

As stated, there is an interconnection between gut microbiota, hypertension, and kidney disease (81). CKD is linked to hypertension and is featured by immune dysregulation and metabolic disorder due to the gut dysbiosis (133).

It is reported that the prevalence of hypertension increases gradually by increasing BMI from ≤5th (2%) to ≥95th (11%) (134). Moreover, higher fecal SCFA levels are associated with hypertension, gut dysbiosis, obesity, and cardiometabolic disease (84), which act to damage the kidney (132, 135, 136).

The reciprocal crosstalk between hypertension and kidney disease seems to be linked to the presence of the olfactory receptor (Olfr78) in the renal juxtaglomerular apparatus, which participates in the secretion processes of renin in response to the intestinal SCFAs (58, 137). In this regard, it was demonstrated that when using antibiotics confining the gut microbiota potential, blood pressure is increasing in *Olfr78* knockout mice (58).

Shifting in microbiome profile was observed in patients with pulmonary hypertension (PAH) (138) compared to a reference cohort. Intestinal synthesis of arginine, proline, and ornithine was increased as well as TMA/TMAO and purine metabolism in PAH proving a shifting of the gut microbiota (139). TMA should be metabolized in TMAO accelerating atherosclerosis (139). Unlike in the reference cohort, butyrate- and propionate-producing bacteria were shown in increasing numbers (139).

#### Gut Kidney Microbiome Axis and Pregnancy-Related Complications

Numerous studies support the involvement of human microbiota and microbial translocation in preeclampsia (PE) (140–143). A meta-analysis of epidemiologic studies indicated that any viral or bacterial infection relates to a higher risk of PE (two-fold) (91).

The placental microbiome exerts regulatory role in normal pregnancy (144). A beneficial interaction between active maternal immune system and human microbiome, gut, kidney, and placental, leads to pregnancy complications such as PE or fetal rejection (145, 146). The impact of microbial translocation (34, 147) into amniotic cavity and placenta (148, 149) is still unclear. It was proposed that bacterial translocation contributes immune cells, which could transport microorganisms by APCs (150).

Moreover, the bacterial translocation is carried by hematogenous dissemination (151). The placental microbiome of PE women often consists of *Prevotella intermedia, Treponema denticola, Porphyromonas gingivalis, Actinobacillus actinomycetemcomitans, Fusobacterium nucleatum, Mycoplasma* species, and *Tannerella forsythensis* (152, 153).

Dysbiosis leads to Immunological and metabolic shifts initiating PE pathophysiology (154, 155). The increased estrogen levels in pregnant women lead to increased deposits of glycogen in the vaginal epithelium, which provides a better substrate for the growth of microorganisms, consequently bacterial translocation (154, 155). Moreover, bacterial contamination of placenta could impact upon endothelial permeability (155). As stated, *F. nucleatum* usually presented in oral cavity could spread hematogenous to the placenta and alter the vascular endothelium permeability (156). Yet, high permeability allows colonization by pathogenic organisms, such as *E. coli* (156). The increase of placental bacterial load promotes neutrophil migration, activation, and formation of neutrophil extracellular traps (NETs) (157). Recent data indicated that NETs stimulate the coagulation pathways through elevated tissue factor expression, red blood cell adhesion, and platelet activation (152, 158). Activation of the coagulation pathways is implicated in pathogenesis of PE and leads to multiorgan damage (159).

Furthermore, gut microbiome dysbiosis leads to gestational diabetes, which is an independent risk factor for PE (160, 161). In addition, it was shown that bacterial burden was correlated to other risk factors of PE, such as hypertension and proteinuria (162).

### Therapeutic Approaches

Restoring the balance of the intestinal microbiota seems to be the cornerstone for improving gut dysbiosis that leads to immunological dysfunction, inflammation, and kidney disease.

Diet is important in the shaping of the intestinal microbiota (163–165). In patients with severe CKD, strict dietary restrictions are imposed in order to prevent hyperkalemia and oxalate surcharge. Consequently, these dietary restrictions could influence microbiota functions (69). Additionally, those patients are taking systematically phosphate-binding agents in order to limit phosphate absorption (69). Antibiotic intake to treat dysbiosis modifies heavily the intestinal microbiota and its functions (166, 167). It is of note that also a high-salt diet modifies the intestinal microbiota and contributes to the CKD progression (168). Therefore, decreased levels of *Lactobacillus* were found under high-salt diet (168) in tandem with low populations of Th17 lymphocytes. *Lactobacillus* intake may restore Th17 lymphocyte levels (168).

In line with the above, we stated several therapeutic approaches that reduce the uremic toxins (169) and improve the microbiota. Probiotics, prebiotics, and synbiotics were given as adjuvant therapy as the point to the balance of the intestinal microbiota (166, 169). Probiotics not only improve the levels of uremic toxins in blood but also restore the gut microbial balance (169). However, it is of note that their effect is probiotic strain dependent (166) based on the expression of functional biomarkers. Probiotics enhancement of epithelial intestinal integrity impedes pathogen entry and adhesion into the epithelial cells (170).

In this regard, probiotics create usually a mucus barrier or even produce soluble proteins to protect the host (170, 171). Antimicrobial peptides are produced from several probiotics expulsing pathogenic bacteria (172) or act as signaling peptides (173). Moreover, several *Lactobacillus* are able to turn the gut pH in acid as they produce acids (172). Other *Lactobacillus* could interpose on the pathogens gene and reduce their aggressive progression (174) for example *Lactobacillus acidophilus* against the enterohemorrhagic *E. coli* O157:H7 (175).

Engineering of the gut microbiota with probiotics showed glucose homeostasis and reduced inflammation and hepatic steatosis as probiotics could modulate the bile acid and SCFA profiles and LPS production (176–178). Overall probiotics are live bacteria that participate actively in the gut metabolism (179). Last but not least is the effect of probiotics upon immunity and inflammation (166). Probiotics enhance both innate and adaptive immunity and increase production of IgA. Cytokine increase in the serum is observed due to their immunomodulatory response (166).

However, some controversial effects were shown in patients who underwent dialysis receiving probiotics as proinflammatory cytokines and endotoxin levels were found low (180). We suppose that this may be due to the overhydrated state of the dialysis patients that amends the real presented levels (180).

Prebiotics are non-digestible fibers inducing the growth and activity of intestinal bacteria for the balance of the gut microbiota. They are used as food supplements. Prebiotics produce SCFAs, improve the intestinal barrier integrity and function, regulate the inflammation and the immune system dysfunction, and finally modulate the glucose and lipid metabolism (179). Prebiotics given to CKD adult patients as well as to pediatric patients with ESRD showed decrease in serum urea nitrogen concentration and improved the clinical status (80). In this line, a study in patients who underwent hemodialysis showed reduced plasma levels of the uremic toxins when patients were given dietary fibers for at least 6 weeks (180).

As known, synbiotics combine both probiotics and prebiotics possessing combined activity. Synbiotics were given to multiple renal dysfunctions, and they showed a lowering of the urine toxins, which confluence with CKD improvement and delay progression of CKD (181). Improvement of the microbiota was also observed as higher *Bifidobacterium* and lower *Ruminococcaceae* populations were found (181).

We discussed the importance of SCFAs previously. Alternative therapy with SCFAs such as butyrate, acetate, and propionate improves renal function as they are able to lower inflammation, infiltrating immune cells, and apoptotic cells in kidneys (182).

Fecal microbiota transplantation (FMT) was used as an interventional method in patients with recurrent diarrhea when antibiotics fail (183). There is a debate between scientists on its application mainly based in ethical issues and safety. While safety is thoroughly checked through carefully selected donors and strict microbiological control techniques, international consensus is not effective, yet because of the ethical, logistical, and technical issues (183, 184). *Clostridium difficile* infectious diarrhea was alleviated successfully following FMT (185). There are few published data in renal patients treated with FMT. An interesting case is the successful treatment of diarrhea induced by tyrosine kinase inhibitors in patients with metastatic renal cell carcinoma (186).

Scientists showed the effect of an adsorbent therapy (AST-120) on dialysis patients with CKD (187). AST-120 is an orally given carbon adsorbent that adsorbs indole and indoxyl sulfate (IS) in CKD patients (188). Moreover, AST-120 seems to extend the time to the initiation of dialysis (189) as it improves the clinical image of the patient. The attenuation of the chronic renal failure by reducing proteinuria and oxidative stress was observed (189), as improvement of the tubular injury was effective. However, most studies on AST-120 are originating from Japan, and little knowledge is gained from international scientist community.

Last but not least, we state here the modulation of the intestinal microbiota following kidney transplantation (183, 190).

## Conclusion

In the present review, we aimed to shed light in the crosstalk between human gut microbiome and kidney disease. Intestinal dysbiosis leads to microbiota shifts including unbalance of the normal intestinal microbiota, metabolic disarrangements, inflammation, immunosuppression, and accumulation of uremic toxins, which lead to the gradual kidney failure.

The hygiene hypothesis seems to be effective since early intestinal colonization especially by beneficial bacteria in newborns predispose to a better health status and offer subsequent protection from many different types of diseases. New therapeutic strategies for restoration of the imbalanced microbiota involve probiotics, prebiotics, synbiotics, and adsorbent therapy but also the questionable fecal transplantation therapy.

Microbial-modulating approaches seem to be the gold standard for prophylaxis and therapy especially after the failure of multiple courses of antibiotics given also their low cost. However, the implementation of such therapies must be applied with attention following strict selection criteria, efficacy, and safety issues.

We highlight here the need to proceed to more clinical investigations in large samples of renal patients, as well as basic research for enriching our knowledge on the kidney–gut axis and undertake effective and safe therapeutic approaches.

## Author Contributions

ES: conceptualization, writing, and editing. TK and KK: formal analysis and writing. GR: writing and investigation. CV: resources and writing. CT: writing and editing. EB: supervision, original draft preparation, and editing. All authors contributed to the article and approved the submitted version.

## Conflict of Interest

The authors declare that the research was conducted in the absence of any commercial or financial relationships that could be construed as a potential conflict of interest.
